# Assessing longitudinal pathways between maternal depressive symptoms, parenting self-esteem and infant temperament

**DOI:** 10.1371/journal.pone.0220633

**Published:** 2019-08-05

**Authors:** Lea Takács, Filip Smolík, Samuel Putnam

**Affiliations:** 1 Department of Psychology, Faculty of Arts, Charles University, Prague, Czech Republic; 2 Institute of Psychology, Czech Academy of Sciences, Prague, Czech Republic; 3 Department of Psychology, Bowdoin College, Brunswick, Maine, United States of America; Pennsylvania State University, UNITED STATES

## Abstract

**Background:**

Previous studies of relations between parenting self-concepts, parental adjustment and child temperament have been ambiguous regarding the direction of influence; and have rarely followed families from pregnancy through the first year of life. The current study examines change and stability in maternal depressive symptoms, parenting competences and child temperament through the perinatal period until nine months postpartum.

**Methods:**

Czech mothers (N = 282) participated at three time points: the third trimester of pregnancy (Time 1), six weeks (Time 2) and nine months postpartum (Time 3). Questionnaire data concerned depressive symptoms (T1, T2, T3), maternal parenting self-esteem (T1, T2) and sense of competence (T3), and child temperament (T2, T3). A path model was used to examine concurrent and longitudinal relations between these variables.

**Results:**

The analyses indicated longitudinal stability of all constructs, as well as concurrent relations between them. Longitudinal relations supported child-to-parent, rather than parent-to-child, effects: child difficult temperament predicted decreases in perceived maternal parenting competences, but maternal variables did not predict change in infant temperament. In addition, we observed weak mutual relations between maternal depression levels and parenting competences, such that maternal depression diminished perceived parenting competences that in turn contributed to higher levels of depression.

**Conclusion:**

Mothers’ confidence in their ability to parent is influenced by their experience with a difficult infant and by their depressive symptoms during the child’s first year of life. Depressive symptoms are, in turn, aggravated by mothers’ low perceived competences in the parenting role.

## Introduction

For decades, research has indicated a relationship between child temperament and maternal psychological characteristics such as depressive symptoms. Such findings have been interpreted as evidence for both parent-to-child [1–6] and child-to-parent effects [7–11]. Similarly, although maternal depression and child temperament have been linked to self-concepts about parenting [12, 13], the directionality of these relationships has been ambiguous. Relationships between maternal psychological status, parenting competences and child temperament are complex and the direction of the effects between them requires further investigation. The current study employs a longitudinal design spanning the perinatal period to enhance inference regarding the degree to which child temperament, maternal self-esteem and depressive symptoms shape one another during the transition from pregnancy to infancy/parenthood.

Depression is a common complication of the perinatal period, with 6–38% of women experiencing depression during pregnancy and 8–23% suffering from depression postpartum [14]. Maternal depression is considered a risk factor for child outcomes, especially as regards cognitive and psychomotor development [1, 15, 16]. However, the studies investigating relations between maternal depressive symptoms and children’s socioemotional characteristics have yielded inconsistent results. Some authors found that maternal depressive symptoms were associated with elevated levels of difficult temperament and child emotional and behavioral problems [1, 12, 17–20], while others evidenced no such association [21–23].

Only a few studies have jointly considered the effects of pre-partum and postpartum depressive symptoms on child emotional and behavioral characteristics. Some investigations have suggested that temperamental and behavioral difficulties are related more strongly to postnatal than prenatal exposure to maternal depression and emotional stress [3, 24, 25]. These findings are consistent with those of Bagner et al. [26], who concluded that the first year of life may be regarded a “sensitive period” in terms of the effects of maternal depressive symptoms on child’s emotion regulation. Nevertheless, Rouse and colleagues [27] reported that depressive symptoms in the antenatal but not the postpartum period predicted infant negative affectivity, while Lusby et al. [28] concluded that prenatal and postpartum depressive symptoms interacted to predict infant’s electroencephalograph (EEG) asymmetry scores, which have been linked to higher levels of inhibited temperament in children [29].

The potential risk pathways are presumed to be different for depressive symptoms experienced in pregnancy and postpartum. Whereas depressive symptoms in pregnancy may have a programming effect on the fetus by affecting placenta function and exposing the fetus to higher cortisol levels [24], the effect of postpartum depression is considered to be mediated by reduced maternal sensitivity and responsiveness to her child and impaired mother-child interactions [25, 30]. Moreover, mothers who are emotionally stable and self-confident may evaluate difficult aspects of their children’s temperament less severely than depressed mothers. Indeed, the depression-distortion hypothesis has been proposed as an explanatory mechanism for the link between maternal depression and children’s maladjustment, suggesting that depressed mothers tend to overrate their children’s behavior problems [31].

Depression is often accompanied by symptoms of anxiety that are considered independent predictors of child temperamental and behavioral difficulties [3, 21]. Both maternal trait and state anxiety have been linked to development of temperament attributes, including negative emotionality and poor attentional regulation, that have frequently been associated with behavior problems [3, 21, 32, 33]. High levels of anxiety experienced in late pregnancy have been shown to predict behavioral and emotional problems in children independently of maternal anxiety symptoms postpartum [34, 35].

The link between maternal anxiety and infant temperament development may be explained by several mechanisms that are similar to those related to perinatal depression. First, anxious women are more prone to experiencing stress during pregnancy, which may have a programming effect on the fetus [36]. Second, trait anxiety may be associated with less optimal parenting practices, which in turn lead to behavioral difficulties in children [32]. Third, given the heritability of personality traits, children of anxious mothers may inherit personality characteristics that manifest themselves as aspects of difficult temperament.

In contrast to investigations assuming that the link between maternal psychology and child temperament flows from mother to child, some researchers have explored the opposite causal direction. For example, Cutrona and Troutman [10] found that difficult temperament in children affected maternal depression both directly and indirectly, with the impact of temperament mediated through parenting self-efficacy. More recent studies investigating child-to-mother effects have found support for the proposal that child temperament contributes to later maternal mood disorders: two cross-sectional studies indicating relations between infant difficult temperament and maternal depressive symptoms were interpreted as evidence of child-to-mother effects in the early postpartum period [8, 9]. One study that controlled for earlier levels of maternal symptomology through multiple regression arrived at the conclusion that child mood at 6 months did not affect maternal depression at 24 months [37]. Two others used cross-lagged path-models to examine associations between child characteristics and maternal depression: Allman et al. [38] found children’s negative affectivity to predict increases in maternal depression between child ages 3 and 9 years, whereas Forbes et al. [39] reported that child EEG right hemisphere asymmetry at age 5 years was associated with elevated levels of maternal depressive symptoms one year later, also finding effects of maternal symptoms on later child emotionality.

Mothers’ impressions of their own competence in parenting appear to play an important role in the relationship between maternal depressive symptoms and child temperament. For instance, mothers who experienced depressive symptoms postpartum reported higher parenting stress [12, 40] and lower parenting satisfaction and self-efficacy [41, 42]. Furthermore, low parenting self-efficacy was, in turn, found to exacerbate the symptoms of depression [4, 8, 10]. A similar pattern is evident for the relationship between maternal perceptions of their own competence in parenting and child temperament: maternal parenting self-efficacy beliefs were found to affect child temperamental characteristics [43, 44], while other authors concluded that perceived difficulty of temperament in children lowers mothers’ parenting self-efficacy [4, 42, 45].

### The aim of the current study

Previous studies on the associations between maternal mood, parenting self-esteem, and child temperament have often adopted cross-sectional designs [8, 9, 33], which limits inference regarding causality between the variables under investigation. The few studies that have employed longitudinal designs have typically used multiple regression analysis [3, 21, 37, 46, 47], which could have resulted in inadequate estimates of the longitudinal relations. To our knowledge, only two studies have applied cross-lagged path models to longitudinal relations between maternal symptomatology and children’s emotional behavior [38, 39]. To explore longitudinal relations between maternal depressive symptoms, parenting competence, and infant temperament, the present study used structural equation modeling with observed variables (path analysis), including data on infant temperament from two time points and on the remaining variables from three time points. This way, a cross-lagged model could be fit that took into account potential mutual influences between all three variables. We were interested in the predictive relations, and anticipated that high levels of difficult child temperament would be linked to high depressive symptomology and low perceived parenting competence in mothers, with depression symptoms and low perceived competence linked to one another. We did not, however, have strong hypotheses regarding causal direction. Because different prior studies have implicated each of these variables as predictors of change in one another, the current investigation offered an opportunity to clarify the direction of influence between infant temperament, maternal depressive symptoms and self-concepts about parenting.

## Materials and methods

### Study design and participants

Pregnant women were recruited from five maternity hospitals in the Vysočina Region of the Czech Republic (Havlíčkův Brod, Jihlava, Třebíč, Pelhřimov, Nové Město na Moravě) from October 2013 to September 2014. The Vysočina Region is a mostly rural area with lower income compared to the national average. Maternity hospitals from which the sample was recruited were prevailingly smaller-size units. The maternity health care system in the Czech Republic is a highly medicalized, obstetrician-led model of care, with obstetricians typically responsible for perinatal outcomes, and without an independent midwifery profession. The rate of Cesarean sections is approximately 27% in the Czech Republic. Women usually take a long-term paid maternity leave in the Czech Republic (up to four years) to care for their child. The majority of mothers are on a maternity leave during the first year of their child’s life.

At recruitment, women were approached by the hospital staff and invited to participate. They were given both oral and written information about the study and asked to sign an informed consent if they agreed to participate. The study was approved by the Ethics Committee of the Jihlava Hospital, which is responsible for the Vysočina Region where the study took place.

The data were collected via questionnaires administered at three time points: third trimester of pregnancy (T1), six weeks (T2) and nine months postpartum (T3). Data concerning the course of labor and newborns’ health status were extracted from medical records. The Edinburgh Postnatal Depression Scale (EPDS) [48] was applied to assess maternal levels of depressive symptoms at T1, T2 and T3. The Maternal Self-Report Inventory-Short Version (MSRI) [49] was used to assess maternal parenting self-esteem at T1 and T2, while the Parenting Sense of Competence Scale (PSOC) [50] was applied to assess parenting competences at T3. The Infant Characteristics Questionnaire (ICQ) [51] was used to assess child temperament at T2 and T3. Mother-infant pairs were excluded if the pregnancy was multiple; the woman was aged below 18 or above 44 years; Apgar scores of the infants at 10 minutes were 7 or under; the child was born before 37^th^ week of gestation, her/his birth weight was lower than 2500g and/or her/his postnatal stay at the maternity hospital was 11 or more days.

**Fig 1:**
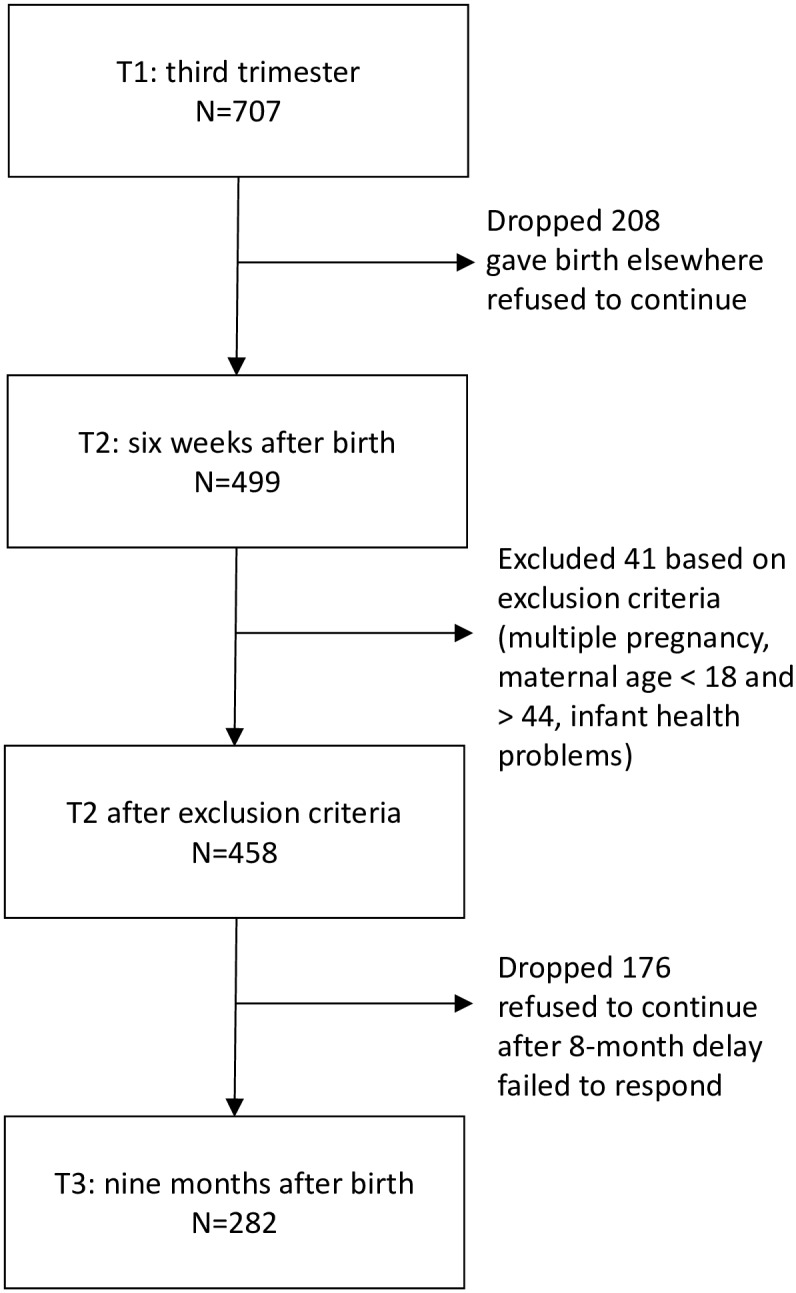
A flow-chart for the sample attrition.

A total of 707 mothers completed the questionnaires at T1. Medical records were available for all of these women. Out of them, 499 completed the questionnaires at T2, and 306 also completed the questionnaires at T3. The decline in response rate from T1 to T2 was expected, as many of the women who visited the maternity hospital for the pregnancy check actually gave birth in a hospital other than the one in which they had their medical check during pregnancy, with some of them giving birth in hospitals outside the Vysočina Region where the study took place. After applying our exclusion criteria, the sample for which we had questionnaires at T1 and T2 consisted of 458 women, 282 of whom also completed questionnaires at T3. The attrition rates and number of women excluded based on our criteria are shown in Fig 1. The characteristics of this final sample, as well as of the women who dropped out the study between T2 and T3, are reported in Table 1. Women who were lost to follow-up at T3 were more often married, more likely to have given birth to a son, and less likely to exclusively breastfeed at six weeks postpartum.

**Table 1:** Characteristics of the sample.

	Final sample^a^		Women who dropped out of study between T2 and T3		
	N = 282		N = 176		Comparison p-value (t-test or Fishers exact test)^b^
Age	30.04	3.95 (18; 44)	29.87	4.34 (19; 42)	0.66
Education					
elementary	1	0.35	3	1.70	0.06
vocational	19	6.74	17	9.66	
secondary	103	36.52	76	43.18	
university	159	56.38	80	45.45	
Parity					
primipara	148	52.48	89	50.57	0.77
multipara	136	47.52	87	49.43	
Marital status					
single	75	26.60	31	17.61	0.03
married	201	71.28	136	77.27	
divorced	5	1.77	8	4.55	
widowed	0	0	1	0.57	
missing	1	0.36	0	0	
Delivery type					
spontaneous	183	64.89	122	69.32	0.15
emergency CS	41	14.54	32	18.18	
planned CS	45	15.96	18	10.23	
operative vaginal	13	4.61	4	2.27	
Child sex					
boy	122	43.26	95	53.98	0.03
girl	160	56.74	81	46.02	
Breastfeeding when leaving hospital					
full	249	88.30	158	89.77	0.87
partial	26	9.22	15	8.52	
none	7	2.48	3	1.70	
Breastfeeding in 6 weeks					
full	238	84.40	132	75.00	0.03
partial	14	5.67	21	11.93	
none	28	9.93	23	13.07	

^a^The sample of women who completed the questionnaires at all three time points and met inclusion criteria.

^b^Continuous quantitative variables were compared using t-tests, categorical variables using the Fisher’s exact test.

### Instruments

#### Maternal prenatal and postnatal depressive symptoms

The EPDS [48] is a 10-item self-report measure for postpartum depression that has also been validated for use with pregnant women [52]. Each item is rated on a 4-point scale from 0 to 3. The total score may thus range from 0 to 30, with higher scores reflecting higher levels of depression.

#### Maternal prenatal and postnatal parenting self-esteem and sense of competence

The MSRI [49] is a 26-item questionnaire, however, 14 items are inappropriate for pregnant women since they are related to experiences with the new-born child. Consequently, a 12-item version was used in pregnancy [53], while the full-item version was used at 6 weeks postpartum. The items are rated on a 5-point scale, such that total scores could range from 12 (26) to 60 (130), with higher scores indicating lower maternal self-esteem.

Because the MSRI is primarily suitable for pregnancy and the early postpartum period, asking mothers how competent they expect to be in their parental role and how they expect their baby to develop *in the future* (for example, *“I am concerned that I will have trouble figuring out what my baby needs”* or *“I have real doubts about whether my baby will develop normally”*), a different scale was chosen to assess parenting sense of competence at 9 months postpartum: the Parenting Sense of Competence Scale (PSOC) [50]. The PSOC is more appropriate for women who have been mothers for a while and are therefore able to assess how competent they consider themselves to be as a parent (for example, *“Considering how long I have been a mother*, *I feel thoroughly familiar with this role*”). The PSOC consists of two subscales (Satisfaction and Efficacy) and includes a total of 17 items rated on a 6-point scale. For the data analysis, the subscales were grouped together in a single measure of parenting self-esteem. The total score could range from 17 to 102, with higher scores suggesting higher parenting self-esteem.

Although the two instruments were designed for use with women who are at different points in their parenting experience, the concepts measured by the MSRI and PSOC are sufficiently similar to be used in a longitudinal study, as they both focus on perceived parenting skills, capabilities, and satisfaction in the maternal role. Both MSRI and PSOC assess whether the mother perceives her maternal responsibilities as manageable, whether she believes she will be able to solve effectively the problems related to caregiving and will know what to do in demanding situations, whether she feels secure and well-prepared for the maternal role, and whether she feels happy and satisfied being a mother. The consistency in the underlying constructs is further confirmed by the stability coefficients in the current study. As shown in the results section, the correlations between MRSI and PSOC scores are similar in magnitude to those between the two administrations of the MSRI.

#### Child temperament

The ICQ [51] is a screening tool for child difficultness, and assesses the degree to which a child is fussy, unadaptable, unpredictable and unenjoyable. The original version of the ICQ contained 24 items, however, with reference to the factor analysis reported [51], only 16 items were applied in this study. Each item was rated on a 7-point scale, with all item scores combined to form a single score indicating a more difficult temperament.

### Statistical analysis

The key results in the present paper are reported using path analysis, i.e., a structural equation model examining the relations between observed, not latent, variables. The approach was used to fit a cross-lagged panel model examining the mutual predictive influences between different variables. The analysis was executed using the R package lavaan [54], using the full-information maximum likelihood (FIML) fitting method. The modeling strategy was empirical and data-driven, constrained with logical relations between the variables. The variables included in the model were maternal depressive symptoms (EPDS), parenting self-esteem (MSRI, PSOC) and difficult temperament in children (ICQ). We built a model for which the three longitudinally observed variables were connected with autoregressive paths, regressing the later measurements on the previous ones. In variables that were measured three times, the initial model also included regressions of the final measurement on the first measurement. The model included cross-lagged regressive paths that examined the effects of variables in the previous rounds on different variables in subsequent rounds. In addition to the regressive paths, correlated residuals between different variables within the same time point were included.

All variables were controlled for four covariates, all treated as binary variables: parity (0 vs. 1 or more), pregnancy complications (diabetes, hypertension) or birth complications (blood loss ≥ 1000 ml, postpartum laparotomy, severe perineal rupture, surgery in the 3^rd^ or 4^th^ stage of labor) (absent vs. present), birth mode (spontaneous vaginal vs. operative), and education (non-university vs. university). The initial model was saturated, so its fit could not be evaluated. After fitting the original saturated model, the non-significant paths and correlations were removed as described in the Results section. Evaluations of the acceptability of fit were based on the RMSEA.

## Results

### Prevalence of depression

A total of 9.45%, 8.54% and 10.81% women scored above cutoff score of 12 on the EPDS in the third trimester of pregnancy, six weeks postpartum and nine months postpartum, respectively (calculated from all available data for each time point).

### Preliminary analyses

The descriptive statistics and Cronbach’s α’s for all questionnaires used (EPDS, MSRI, PSOC and ICQ) are presented in Table 2. The correlations among major variables are shown in Table 3. Maternal pre- and postpartum depressive symptom levels were moderately correlated, as were temperament scores at 6 weeks and 9 months postpartum. No significant correlation between antenatal depressive symptoms and child temperament was observed, but mild correlations were found between maternal postpartum depressive symptoms and child temperament, both concurrently and longitudinally. Maternal prenatal and postnatal self-esteem were moderately correlated. Maternal self-esteem measured in pregnancy showed no significant associations with child temperament (neither 6 weeks nor 9 months postpartum), however, significant associations with temperament were observed when maternal self-esteem was measured in the postpartum period. The correlations between maternal depressive symptoms and self-esteem/parenting competences were rather low, except when measured postpartum at the same time point. S1 and S2 Tables in supplemental material show the descriptive statistics of maternal self-esteem and difficult temperament separately for mothers who met and did not meet the clinical cutoff for depression according to EPDS.

**Table 2:** Descriptive statistics for the EPDS, MSRI, PSOC and ICQ.

	N	Mean	SD	Median	Min	Max	Cronbach’s α
EPDS (pregnancy)	274	6.24	4.57	0	0	21	0.84
MSRI (pregnancy)	278	13.61	4.86	8	8	36	0.77
EPDS (6 weeks postpartum)	275	6.37	4.54	0	0	21	0.86
MSRI (6 weeks postpartum)	259	30.81	11.72	11	11	68	0.86
EPDS (9 months postpartum)	270	5.12	4.44	0	0	22	0.85
PSOC (9 months postpartum)	251	21.63	9.16	2	2	52	0.83
ICQ (6 weeks postpartum)	266	40.88	9.87	18	18	68	0.82
ICQ (9 months postpartum)	254	41.3	10.65	17	17	72	0.84

**Table 3:** Correlations among major variables (EPDS, MSRI, PSOC, ICQ) in the subsample used for path analysis (n = 282).

	MSRIpreg	MSRI6w	EPDSpreg	EPDS6w	EPDS9m	Temp6w	Temp9m	PSOC9m
MSRIpreg	-	-	-	-	-	-	-	-
MSRI6w	0.52	-	-	-	-	-	-	-
EPDSpreg	0.23	0.32	-	-	-	-	-	-
EPDS6w	0.17	0.50	0.57	-	-	-	-	-
EPDS9m	0.19	0.38	0.49	0.55	-	-	-	-
Temp6w	0.11	0.40	0.20	0.27	0.26	-	-	-
Temp9m	0.03	0.22	0.11	0.21	0.28	0.52	-	-
PSOC9m	0.41	0.51	0.30	0.38	0.48	0.39	0.38	-

Note: The N’s for individual cells range from 251 to 275. The critical value for p = 0.001 is below 0.21; for p = 0.01 below 0.16; preg–pregnancy; 6w –six weeks postpartum; 9m–9 months postpartum.

### Path analysis

**Fig 2:**
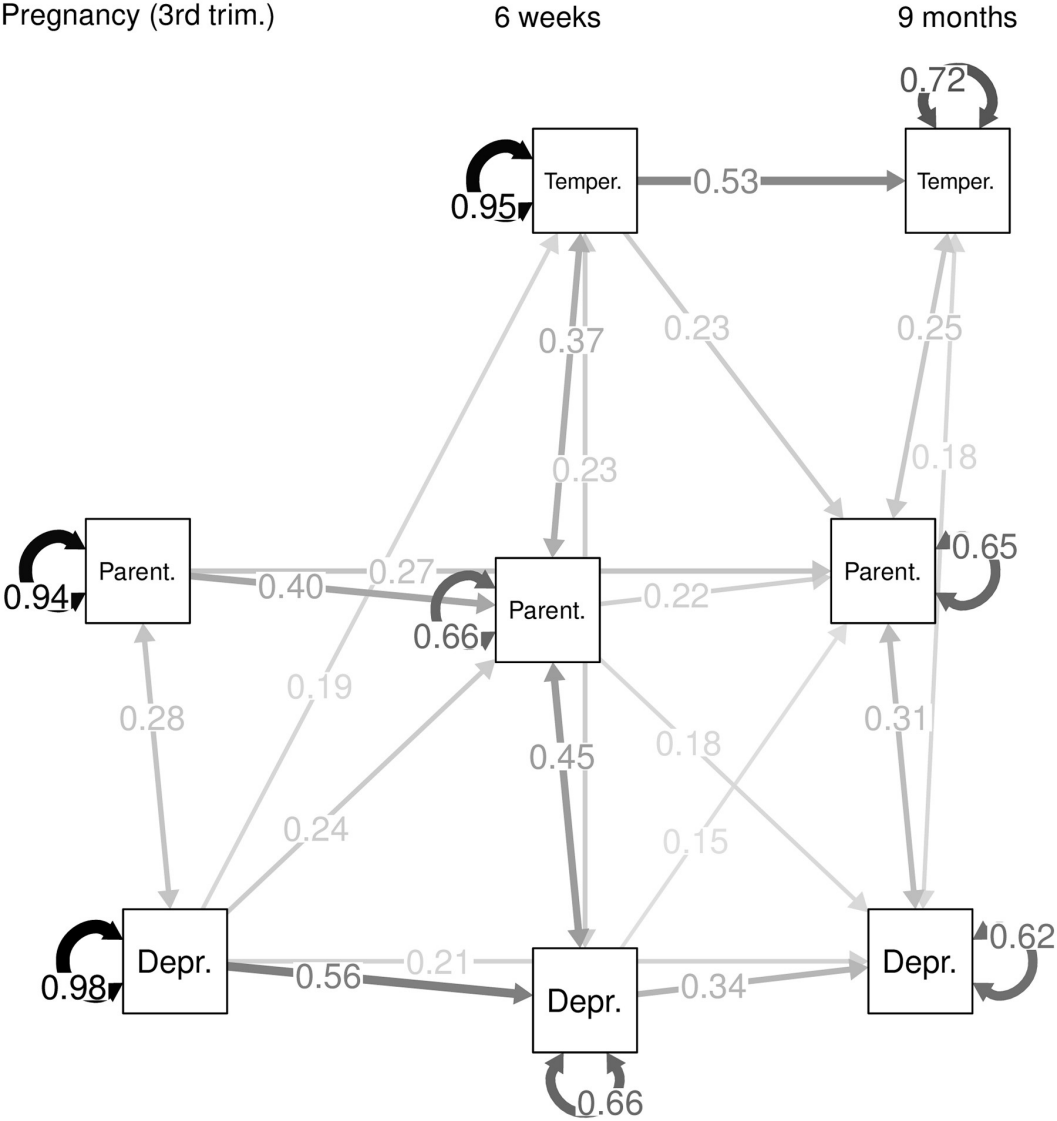
Path diagram of the final model.

After fitting the initial saturated model, a subsequent model was fit for which all paths with p-values > 0.2 were removed. This model showed good fit (RMSEA < 0.1, χ2(28) = 20.66, p = 0.84). Most standardized paths or correlations in this model were significant at alpha = 0.05, except for three regression paths of the variables of interest on covariates, for which the p-values were 0.07, 0.08 and 0.13. The path diagram of the final model including the estimated correlations, standardized regression coefficients and residuals, is presented in Fig 2 (covariates omitted).

The final path model includes correlated residuals between all variables at each time point (EPDS and MSRI in pregnancy, EPDS and MSRI, EPDS and ICQ, MSRI and ICQ 6 weeks postpartum, EPDS and PSOC, EPDS and ICQ, PSOC and ICQ 9 months postpartum). There are significant predictive relations between variables measuring the same concept at different time points (child temperament, maternal self-esteem, and maternal depressive symptoms), which confirms the stability of these reports regarding mothers’ psychology and their perceptions of their infants.

Of main interest were the cross-lagged correlations between different variables. The key finding of interest is that, after the child’s birth, children’s difficult temperament was associated with diminished maternal sense of competence, but there were no effects of maternal variables on difficult temperament. Prenatal depression levels were predictive of higher ratings of difficult temperament at 6 weeks postpartum. However, neither maternal depression nor maternal sense of competence were associated with change in temperament from 6 weeks to 9 months. With regards to maternal depression and maternal sense of competence, prenatal depression values had a negative effect on maternal competence six weeks postpartum, and each of the variables at six weeks postpartum affected the other at nine months postpartum.

## Discussion

The current study used cross-lagged analysis to explore longitudinal connections between maternal depressive symptoms and parenting self-esteem, assessed in the third trimester of pregnancy and then again twice in the postpartum period, along with perceived child temperament measured twice in the first postpartum year. This design and statistical strategy allowed us a greater degree of inference regarding direction of effects between these constructs than previous studies that used cross-sectional design or regression analyses of longitudinal data. Our results supported the view that child-to-parent effects, more so than parent-to-child influences, characterize the relations among maternal psychological status, parenting competences and child temperament in the postpartum period.

We found evidence for child effects on mothers’ perceived competence, but no longitudinal effects of parenting self-concept on child temperament. Although depressive symptoms in pregnancy predicted infant temperament six weeks postpartum, the effect of prenatal depression levels on infant temperament did not persist beyond this period, nor did postnatal maternal depression contribute to change in infant temperament. Regarding links between aspects of maternal psychology, prenatal depression had a detrimental effect on maternal feelings of competence in the second month following the child’s birth. Over the subsequent several months, the relation became mutual, such that lowered parenting competence contributed to depression, and continuing depression further diminished the perceived parenting competence. Our findings thus confirmed the independent roles of child characteristics and maternal symptomology in shaping mothers’ views of their own parenting competences and the existence of mutual relations between depression and parenting competences.

Previous studies have conceptualized difficult temperament as both a contributor to lower parenting self-efficacy [42, 45, 53], and a result of poor perceived parenting skills [43]. Few, however, have applied cross-lagged analyses to longitudinal data to investigate change in these across time. The current findings in favor of child effects on parenting, but not the reverse, are consistent with those obtained by Cutrona and Troutman [10] and Gross et al., [4], but contradict the report of Verhage et al. [44], who concluded that maternal parenting self-efficacy affects mothers’ perceptions of child temperamental characteristics, and not the other way around. A potential explanation may lie in the questions and constructs included in the measures of temperament in these studies. The current study, along with others evidencing child to parent effects [4, 10], used instruments explicitly focused on the concept of infant difficultness, measuring this construct through general questions regarding parents’ perspectives on their children, which are answered with respect to the degree of difficulty these characteristics present to the parent (e.g., *“How easy or difficult is it for you to calm or soothe your baby when he/she is upset*?*”*, answered with responses ranging from *very easy* to *difficult*). In contrast, the Infant Behavior Questionnaire (IBQ) [55], used by Verhage et al. [44] in a study indicating parent to child effects, assesses more specific dimensions, using items tapping infants’ concrete behaviors in specific situations and time frames (e.g., *“When having to wait for foods or liquids during the last week*, *how often did the baby cry loudly*?*”*, answered with responses ranging from *never* through *half the time* to *always*). Although the variance in responses to both types of instrument presumably contains both objective and subjective components [56], these components may differ across instruments. It may be the case that the generalized and parent-centered questions of the ICQ and similar measures emphasize aspects of the parenting experience that serve to shape mother’s perspectives regarding their own effectiveness, whereas the concrete behaviors assessed through the IBQ reflect tendencies in children’s responses–or perhaps biases in parental perceptions—that are open to influence by parenting actions associated with self-confidence.

Our results suggest that there are reciprocal relations between maternal depression symptoms and perceived parenting competences. These relations are not very strong but they are in line with previous research that had demonstrated depressive symptoms in the perinatal period to be linked to higher parenting stress [12, 40, 57] and lower parenting satisfaction and self-efficacy [41, 42]; and with prior studies showing low parenting self-efficacy to be, in turn, associated with higher levels of depression [4, 8, 10]. As such, our results contribute to a body of evidence showing detrimental effects of depression on mothers’ ability to cope with difficult infants. Such findings speak to the importance of interventions aimed at diminishing rates of postpartum depression [58, 59] and suggest that components intended to prevent depression from impacting parenting efficacy, or efforts to improve parenting confidence as a route to diminishing maternal depression, may be useful.

Our results further suggest that depressive symptoms in pregnancy predict maternal assessment of infant temperament in the early postpartum, and concurrent links between depression and temperament were evident in our correlational analyses. There were, however, no postnatal cross-lagged paths between depressive symptoms and temperament. Whilst difficult infant temperament may affect maternal parenting competences, it did not appear to exacerbate mothers’ depressive symptoms in the current sample. These findings are in agreement with others reporting the effects of prenatal depressive symptoms on child behavior [27], those indicating concurrent connections between maternal depression and child temperament [3, 10, 47], and those demonstrating no longitudinal effects of infant temperament on maternal depression [37, 57]. They are, though, inconsistent with those evidencing mother to child or child to mother effects in the postpartum period [3, 8–10, 38, 39, 47, 60].

Important differences exist between these prior studies and the current work. Waerden et al. [60] assessed the link between child behavioral problems and maternal depression trajectories from pregnancy to five years postpartum, and Allmann et al. [38] examined temperament at age three in relation to depression six years later. The effect of infant temperament on maternal mental health may build steadily over time, such that the relatively brief span in the current study was insufficient to substantially change mothers’ wellness, in comparison to the pervasive effect of caring for a challenging child over several years. Related to this possibility, mothers’ ratings of their child’s temperament were only modestly stable in the current study, relative to the degree of consistency typically observed in the toddler and preschool periods. The relative flexibility of infant behavior (and/or mothers’ subjective experience of it) may have limited the possible effect of temperament upon mothers’ depression symptoms. Forbes et al. [39] reported a cross-lagged model showing effects of maternal depressive symptoms on child behavior a year later, but did not rely on maternal questionnaire reports of child temperament, instead assessing child affectivity using observation of mother-child pairs during interaction tasks. It is possible that maternal behavior during these tasks influenced the behavior of children, with more depressed mothers influencing their children’s affect through emotional contagion. While our analysis assessing cross-lagged effects, controlling for stability of both depression and temperament in the first year, is thus an important contribution to understanding of the long-term relations between child temperament and mothers’ psychological well-being, further studies varying by child age and temperament measurement will shed additional light on such effects.

Maternal depressive symptoms in pregnancy predicted depressive symptoms six weeks postpartum, and both pre-partum and early postpartum depressive symptoms predicted depression levels nine months postpartum, suggesting that they have independent effects and contribute additively to later depression. Similar results were obtained for maternal self-esteem, however, the association between pre-partum self-esteem and self-esteem nine months after childbirth is stronger than that between self-esteem six weeks and nine months postpartum. This might seem surprising, given that the latter association is closer in terms of time. The unique nature of the early postpartum period may provide an explanation. Recovery from delivery, adjustment to new family dynamics, and related sources of stress may play an outsized role in determining maternal self-esteem at 6 weeks postpartum, whereas maternal confidence measured during pregnancy and late in the first year of a child’s life may reflect a more pervasive array of cognitions regarding parenting.

There are limitations that should be considered when interpreting our results. Primary among these is that mothers reported on both their own psychological status and their children’s temperament. This limitation of relying on a single source is particularly profound with respect to the interpretation of concurrent effects, as reports of mothers’ feelings regarding parenting, their symptomology, and their impressions of their children may all be influenced by a common element of maternal personality. With respect to cross-lagged effects, as suggested above, mothers’ perceptions of their child being difficult, rather than the child’s behavior, per se, may be critical in shaping maternal adjustment. Although such an interpretation does call to question the actual impact of children’s objective characteristics on their mothers’ well-being, it does speak to the importance of mother’s perspectives on their infant, suggesting potential routes for treatment. Additional research that includes multiple different measures of temperament, including behavioral observations as well as more recently developed questionnaires that incorporate fine-grained attributes involving different forms of positive and negative affect, and of capacities for self-regulation (e.g., the Infant Behavior Questionnaire-Revised, [61]), would generate important insight regarding exactly what about mothers’ ratings of child temperament leads to greater depression.

Another limitation of this study is the composition of the sample. Data of 458 women were available initially, however, only 282 women were included in the final analysis due to attrition over time. Moreover, those women who dropped out the study between six weeks and nine months postpartum differed significantly from those who did not: they were more often married, more likely to have given birth to a son, and breastfed fully less frequently. The relatively high attrition rate, resulting in a higher rate of breastfeeding women in a final sample compared to the initial one, may limit the generalizability of our results, such that they apply most appropriately to mothers who possess the means and motivation to engage in caregiving practices associated with healthy child development.

Given the demographics of the Czech Republic, the women in the sample were ethnically homogenous. Although this does not limit possible generalizations for the Czech population, it may limit the generalizations internationally or to ethnically diverse populations. In addition, half of the participating women had a university degree and about 70% of them were married, which may also limit the generalizability of the results.

In this study, we used a community sample, which resulted in relatively low levels of depressive symptoms. There are no official statistics regarding the prevalence of maternal depression available in the Czech Republic, however, prevalence of postpartum depression was reported to be 12.8% during pregnancy, 11.8% six weeks after childbirth, and 10.1% six months after delivery in the Czech sample of women who participated in the Czech part of the European Longitudinal Study of Pregnancy and Childhood (ELSPAC) from 1991 to 1992 [62]. The authors of the ELSPAC used the same measure to assess depression levels [48], but they applied a somewhat lower cutoff than in our study (10 vs. 12), suggesting that the depression levels in our sample are comparable to those reported previously in similar region.

An additional limitation concerns inconsistency in our measures of parenting competences; we employed Maternal Self-report Inventory (MSRI), a measure of maternal self-esteem, in pregnancy and at six weeks postpartum [53], but used the Parenting Sense of Competence Scale (PSOC) [50] at nine months postpartum. This choice of measures was made on the basis of suitability at the different time points, and the scores on the MSRI and PSOC were correlated over time, which supports our assumption that they measure related psychological constructs, but this applicability at different time points came at a cost of more “pure” measurement consistency.

In our analyses, we controlled for parity, pregnancy and birth complications, delivery mode, and maternal educational level. However, other important factors, such as social support provided to the pregnant women and new mothers from their partners and families, were not included, which might affect the validity of the presented results. As family support may affect both levels of maternal depression and parenting self-esteem [63–65], those variables should be accounted for in future studies.

A final limitation concerns the empirical modelling strategy that was used. The elimination procedure by which we pruned the saturated model carries the risk of capitalizing on chance, resulting in spurious relations that will not replicate. However, the degree of consistency between our findings and the existing research enhance our confidence that they are replicable.

The strengths of this study lie in its prospective longitudinal design and availability of the data regarding maternal depressive symptoms and self-esteem administered not only in the postpartum period, but already in pregnancy. Moreover, this study is one of few studies employing cross-lagged path analysis on longitudinal data regarding maternal depression, parenting self-esteem and child temperament, which enhances our conclusions about the direction of causality between those variables. Finally, these data were gathered from a relatively understudied population. Because expectations, perceptions and goals regarding children and parenting can differ substantially across cultures [66–68], studies carried out in nations outside of the U.S. and Western Europe hold potential for identifying both common and unique patterns of relations between parenting cognitions and child behavior.

## Conclusions

The current study complements a growing literature regarding the dynamic relations between parental adjustment, thoughts regarding parenting, and child characteristics during the formative months surrounding a child’s birth. Our findings suggest that child temperament influences maternal self-efficacy, and does so beyond earlier confidence in parenting. As sense of competence may shape parenting behaviors, but may be at the same time strengthened by the support of women’s social environment, these results highlight the importance of psychosocial support provided to mothers who perceive their child as difficult.

Because our findings further suggest a mutual effect between depressive symptoms and perceived parenting competence, screening for depression might identify mothers who are most vulnerable, and thus may benefit most, from steps taken to prevent the development of poor maternal self-esteem and less favorable parenting practices. Similarly, interventions tailored to strengthen parenting self-esteem, especially for parents of difficult children, may hold special promise for improving family functioning by enhancing maternal mental health.

## Supporting information

S1 Table Descriptive statistics as a function of (not)reaching the clinical cutoff of 12/13 on EPDS during pregnancy. (DOCX)

S2 Table Descriptive statistics as a function of (not)reaching the clinical cutoff of 12/13 on EPDS 9 months postpartum. (DOCX)
