# Supplementary material for: Assessing longitudinal pathways between maternal depressive symptoms, parenting self-esteem and infant temperament
Source: PLoS One. 2019 Aug 5;14(8):e0220633. doi: 10.1371/journal.pone.0220633 (PMC6681961; doi:10.1371/journal.pone.0220633)
Supplement: S1 Table — (DOCX) [file pone.0220633.s001.docx]

**Supporting information**

**S1 Table. Descriptive statistics as a function of (not)reaching the clinical cutoff of 12/13 on EPDS during pregnancy.**

|  | EPDS (preg) <13 | | | | | EPDS (preg) >= 13 | | | | |
| --- | --- | --- | --- | --- | --- | --- | --- | --- | --- | --- |
|  | Mean | SD | Med. | Min | Max | Mean | SD | Med. | Min | Max |
| MSRI pregnancy | 13.39 | 4.9 | 12 | 8 | 36 | 15.13 | 4.77 | 15 | 8 | 28 |
| MSRI 6 weeks | 30.34 | 11.81 | 29 | 11 | 68 | 35.71 | 10.99 | 34.5 | 11 | 55 |
| PSOC 9 months | 20.94 | 8.71 | 20 | 2 | 48 | 26.54 | 11.46 | 29 | 6 | 52 |
| ICQ 6 weeks | 40.59 | 9.61 | 40 | 19 | 68 | 42.72 | 11.96 | 41 | 18 | 66 |
| ICQ 9 months | 41 | 10.73 | 40 | 17 | 69 | 42.93 | 11.04 | 44 | 27 | 72 |
