# Supplementary material for: Assessing longitudinal pathways between maternal depressive symptoms, parenting self-esteem and infant temperament
Source: PLoS One. 2019 Aug 5;14(8):e0220633. doi: 10.1371/journal.pone.0220633 (PMC6681961; doi:10.1371/journal.pone.0220633)
Supplement: S2 Table — (DOCX) [file pone.0220633.s002.docx]

**Supporting information**

**S2 Table. Descriptive statistics as a function of (not)reaching the clinical cutoff of 12/13 on EPDS 9 months postpartum.**

|  | EPDS (9 months) <13 | | | | | EPDS (9 months) >= 13 | | | | |
| --- | --- | --- | --- | --- | --- | --- | --- | --- | --- | --- |
|  | Mean | SD | Med. | Min | Max | Mean | SD | Med. | Min | Max |
| MSRI pregnancy | 13.55 | 4.86 | 13 | 8 | 36 | 15.82 | 5.05 | 15 | 8 | 28 |
| MSRI 6 weeks | 30.07 | 11.09 | 29 | 11 | 68 | 44.47 | 12.46 | 48 | 25 | 65 |
| PSOC 9 months | 21.01 | 9.09 | 20 | 2 | 52 | 30.53 | 7.6 | 30 | 14 | 43 |
| ICQ 6 weeks | 40.82 | 9.8 | 40 | 18 | 68 | 46.69 | 8.39 | 44.5 | 35 | 66 |
| ICQ 9 months | 40.88 | 10.63 | 40 | 17 | 72 | 46.81 | 11.3 | 47 | 26 | 64 |
